# Parameters associated with therapeutic response using peritoneal dialysis for therapy refractory heart failure and congestive right ventricular dysfunction

**DOI:** 10.1371/journal.pone.0206830

**Published:** 2018-11-19

**Authors:** Noemi Pavo, Rajashri Yarragudi, Heidi Puttinger, Henrike Arfsten, Guido Strunk, Andja Bojic, Martin Hülsmann, Andreas Vychytil

**Affiliations:** 1 Department of Internal Medicine II, Clinical Division of Cardiology, Medical University of Vienna, Vienna, Austria; 2 Department of Internal Medicine III, Clinical Division of Nephrology and Dialysis, Medical University of Vienna, Vienna, Austria; 3 Complexity Research, Vienna, Austria; 4 FH Campus Vienna, Vienna, Austria; 5 Technical University Dortmund, Dortmund, Germany; Scuola Superiore Sant'Anna, ITALY

## Abstract

**Background:**

In patients with refractory heart failure (HF) peritoneal dialysis (PD) is associated with improved functional status and decrease in hospitalization. However, previous studies did not focus on right ventricular dysfunction as an important pathophysiologic component of cardiorenal syndrome.

**Methods:**

In a prospective cohort study PD was started in 40 patients with refractory right HF (with/without left HF). Refractoriness to conservative therapy was defined as persistent right heart congestion/ascites with intensified diuretic treatment and/or ≥2 hospitalizations within 6 months because of cardiac decompensation despite optimal medical treatment, and/or acute renal failure during intensified conservative treatment of cardiac decompensations.

**Results:**

Patient survival was 55.0% at 1 year, 35.0% at 2 years and 27.5% at 3 years. The number of hospitalization days declined after initiation of PD for both cardiac [13 (IQR 1–53) days before vs. 1 (IQR 0–12) days after start of PD, p<0.001] and unplanned reasons [12 (IQR 3–44) days before vs. 1 (IQR 0–33) days after start of PD, p = 0.007]. Using a combined endpoint including survival time of ≥1 year and either improvement in quality of life or decline in hospitalizations we found that patients with extended ascites, higher systolic pulmonary artery pressure, more marked impairment of right ventricular function and tricuspid valve insufficiency, higher residual renal function as well as those who could perform PD without assistance have benefited most from this therapy.

**Conclusions:**

Patients with more pronounced backward failure, less marked residual renal functional impairment and those not depending on assistance for therapy are likely to profit most from PD.

## Introduction

Heart failure with reduced ejection fraction (HFrEF) is the final common path of cardiac diseases and is associated with low quality of life and high mortality. Three components predict outcome in end-stage HFrEF. First, decreased kidney function and worse diuretic response are independent predictors of mortality [1, 2]. Second, repeated hospitalizations due to cardiac decompensation (mainly volume overload based on diuretic resistance) are associated with decreased patient survival. Finally, right ventricular dysfunction (RVD) accompanied by fluid redistribution to right heart related organs is regarded as the last sequela of the disease. This phenomenon which in the current concept of cardio-renal syndromes is called backward failure has been supported by several previous papers. In experimental studies increase of renal venous pressure (RVP) by renal vein ligation leads to an immediate decrease in blood flow and estimated glomerular filtration rate (eGFR) accompanied by an increase in aldosterone and renin levels, effects which are reversible again after the decrease of RVP [3]. Accordingly, in the clinical setting, venous congestion due to backward failure and the increase of intraabdominal pressure markedly contribute to the impairment of kidney function and consecutively outcome in patients with cardio-renal syndrome [4]. Conversely, the reduction of intraperitoneal pressure and decongestion results in an improvement [5]. However, decongestion of the dependent compartments of the right heart can only rarely be achieved by diuretics. Similarly, studies focusing on extracorporal ultrafiltration in patients with acute decompensated heart failure reported controversial results [6–8]. In contrast, peritoneal ultrafiltration/peritoneal dialysis (PD) enables gentle continuous fluid removal as well as direct continuous removal of ascites outside of an intensive care setting. Therefore, intuitively, PD may be especially interesting for patients with decompensated RVD. During the last 75 years several authors reported that in patients with refractory HFrEF PD is associated with improvement of functional status and a reduction in hospitalization [9]. However, most of these studies were retrospective and disease severity was mainly defined by physician judgment. The importance of PD in end-stage heart failure, but especially the limitations in knowledge, were recently highlighted by a position paper of the Heart Failure Association [10]. Especially no investigation focused on patients with RVD as the main component of cardiorenal syndrome. We, therefore, aimed to focus on patients with RVD in a prospective long-term cohort study.

## Materials and methods

### Patient population

This is a prospective cohort study enrolling patients with refractory RVD in whom PD treatment was initiated. The study protocol was approved by the local ethics committee of the Medical University of Vienna (EK 334/2008) and carried out in accordance with the Declaration of Helsinki. The first patient was enrolled in January 2009, the last patient finished the study in July 2016. All included patients had to be at least 18 years of age and provided written informed consent to study participation according to GCP and Declaration of Helsinki guidelines. RVD was documented by echocardiography and clinically by signs of edema and/or liver dysfunction and/or ascites. Refractoriness to conservative therapy was defined when at least one of the following criteria was present: 1) persistent right heart congestion/ascites despite treatment with at least 160 mg furosemide and optimal medical therapy (OMT) 2) occurrence of acute renal failure during intensified conservative treatment of cardiac decompensation 3) repeated hospitalizations (≥ 2 hospitalizations within 6 months) because of cardiac decompensation despite OMT [11]. OMT was defined by maximum dosages in accordance to the current guidelines or a repetitive failure of up-titration based on a mean blood pressure below 60mmHg (for RAS antagonists), a heart rate below 55 bpm (for beta-blockers) and a serum potassium >5.5 mmol/L (for mineralocorticoid receptor antagonists). Echocardiographic examinations were performed using commercially available equipment (Vivid 5 and Vivid 7, GE Healthcare, and Acuson Sequoia, Siemens). Cardiac morphology was assessed using diameters and volumes in 4- and 2-chamber views. Assessment of left ventricular function (LVEF) and right ventricular function was performed by semi-quantitative assessment by experienced readers using multiple acoustic windows and graded as normal, mild, moderate or severe. Additionally, left ventricular ejection fraction was calculated using the biplane Simpson method according to guideline recommendations [12]. Right ventricular function was quantified by fractional area change (FAC) and the tricuspid annular plane systolic excursion (TAPSE) from apical 4-chamber views [13]. Mitral and tricuspid regurgitation was quantified by an integrated approach comprising valve morphology, width of the proximal regurgitant jet, proximal flow convergence, and pulmonary venous flow pattern as previously described [14]. Systolic pulmonary artery pressures (sPAP) were calculated by adding the peak tricuspid regurgitation (TR) systolic gradient to the estimated central venous pressure. All patients received dietary counselling at baseline and at least every 6 months (focusing on dietary salt restriction, potassium intake and optimization of protein intake).

### Endpoints

Length of hospitalization stays due to cardiac reasons was investigated as the primary endpoint. To characterize different phenotypes depending on outcome, treatment success at 1 year and overall survival at 2 years were defined as secondary endpoints.

For the primary endpoint, the number of hospitalization days was assessed for the whole observation period after the start of PD and compared to the number of hospitalization days before the initiation of PD for the same time period. For the secondary outcome a combined endpoint termed treatment success was virtually predefined as a survival of at least 12 months combined with an improvement in quality of life (defined as an improvement of MLHFQ by ≥ 20%) and/or a decline in hospitalization days. A successful bridge to candidacy (heart transplantation or left ventricular assist device (LVAD)) was also regarded as treatment success.

### Parameters

Comorbidities, as diabetes, arterial hypertension, chronic obstructive pulmonary disease, peripheral artery disease and cerebral artery disease, were assessed. Routine laboratory parameters including creatinine and N-terminal B-type natriuretic peptide (NT-proBNP), were determined in the central laboratory of the Medical University of Vienna according to the laboratory´s standard procedure. Furthermore, eGFR at baseline was calculated using the Modification of Diet in Renal Disease (MDRD) equation. Calculated glomerular filtration rate was equally performed as an average of renal creatinine and renal urea clearance using 24-h urine samples (24-h GFR). Normalized protein catabolic rate (nPCR) was calculated using the PD Adequest 2.0 software (Baxter Healthcare, Deerfield, IL, USA). Quality of life (QoL) was measured using the Minnesota Living with Heart Failure Questionnaire (MLHFQ) which provides a score between 0 (best) and 105 (worst) for each patient [15]. The observation period was defined as two years. Additionally, survival data are provided for 3 years.

### Statistics

Regarding the primary endpoint sample size was estimated based on previous papers showing a marked decline in hospitalization days in study populations including ≤ 20 patients. [16–19]. We have doubled the population in order to receive reliable results also in subpopulations (e.g. patients with/without PD success). Continuous data were presented as median and IQR and categorical data as counts and percentages. Medians between groups were compared using the Mann-Whitney-U-test, counts by the Chi-Square test. Variables with repeated measurements were compared using the Friedman-test and the Wilcoxon-test. To investigate the impact of baseline characteristics on 2-year mortality after the initiation of PD univariate and multivariate Cox regression analysis was performed for selected variables mirroring prognosis in heart failure as NT-proBNP, serum sodium and LVEF, kidney function and fluid balance as urinary output and GFR, serum blood urea nitrogen (BUN)/creatinine ratio as marker of neurohumoral activation and fluid status, as well as markers of backward failure as butyryl-cholinesterase (BChE) and ascitic fluid volume. For all tests two-sided p-values lower 0.05 were considered to indicate statistical significance.

## Results

### Baseline characteristics

A total of 40 PD patients were included in our analysis. The median of follow-up was 12.3 months (IQR 3.5–24.0; range 0.03–24.00). Baseline characteristics are shown in *Tables 1–3*. Median age was 65 (IQR 59–70) years, 22.5% of patients were female, 42.5% were diabetic. Median eGFR and 24-h GFR at baseline were 19.4 (10.9–33.9) ml/min/1.73 m^2^ and 9.67 (6.24–19.30) ml/min/1.73 m^2^, respectively. Therefore, the percentage of patients with CKD 5 increased from 32.5% to 62.5% when calculated GFR instead of eGFR was used. Nine of the 40 patients were initially treated with intermittent hemodialysis (n = 6) or continuous veno-venous hemofiltration (n = 3) before the start of PD. However, intermittent extracorporal treatments were associated with repeated hypotensive episodes in all 6 patients whereas the 3 patients on hemofiltration required continuous treatment with vasopressors.

**Table 1:** Baseline characteristics of the peritoneal dialysis patient cohort (n = 40) and comparison of variables for the subgroups with successful treatment and 2 years survival. Continuous variables are given as medians and inter-quartile ranges (IQR), counts are given as numbers and percentages. Variables were compared by the means of the Mann-Whitney-U test or the Chi-square test.

	Baseline (n = 40)	PD Success (n = 18)	No PD Success (n = 22)	p-value	2a Survival (n = 14)	No 2a survival (n = 26)	p-value
Age, years (IQR)	65 (59–70)	65 (60–69)	67 (52–71)	0.882	65 (60–69)	67 (58–70)	0.747
Male gender, n (%)	31 (77.5%)	15 (83.3%)	16 (72.7%)		12 (85.7%)	19 (73.1%)	0.453
BMI kg/m^2^, (IQR)	26.0 (22.5–31.0)	27.6 (24.2–31.2)	25.4 (22.2–31.0)	0.209	26.3 (24.2–33.1)	25.8 (22.4–31.0)	0.440
Heart rate, bpm (IQR)	70 (61–76)	65 (60–70)	74 (62–80)	0.083	63 (58–68)	74 (64–80)	**0.006**
Quality of life, MLHFQ (IQR)	67 (53–81)	62 (44–79)	70 (61–89)	0.284	53 (42–77)	70 (62–86)	0.069
Comorbidities							
Ischemic CMP, n (%)	21 (52.5%)	11 (61.1%)	10 (45.5%)	0.360	10 (71.4%)	11 (42.3%)	0.105
Dilatative CMP, n (%)	16 (40.0%)	6 (33.3%)	10 (45.5%)	0.526	4 (28.6%)	12 (46.2%)	0.329
Stroke / TIA, n (%)	7 (17.5%)	3 (16.7%)	4 (18.2%)	1.000	3 (21.4%)	4 (15.4%)	0.679
PAD, n (%)	9 (22.5%)	4 (22.2%)	5 (22.7%)	1.000	3 (21.4%)	6 (23.1%)	1.000
Diabetes mellitus, n (%)	17 (42.5%)	8 (44.4%)	9 (40.9%)	1.000	7 (50.0%)	10 (38.5%)	0.521
Arterial Hypertension, n (%)	30 (75.0%)	15 (83.3%)	15 (68.2%)	0.464	11 (78.6%)	19 (73.1%)	1.000
COPD, n (%)	9 (22.5%)	3 (16.7%)	6 (27.3%)	0.476	3 (21.4%)	6 (23.1%)	1.000
Intracardiac devices / ECG							
PM, n (%)	5 (12.5%)	2 (11.1%)	3 (13.6%)	1.000	2 (14.3%)	3 (11.5%)	1.000
ICD, n (%)	21 (52.5%)	9 (50.0%)	12 (54.5%)	1.000	6 (42.9%)	15 (57.7%)	0.510
CRT, n (%)	14 (35.0%)	8 (44.4%)	6 (27.3%)	0.327	6 (42.9%)	8 (30.8%)	0.501
Atrial fibrillation, n (%)	27 (69.2%)	16 (88.9%)	11 (52.4%)	**0.018**	11 (78.6%)	16 (64.0%)	0.477
Medication							
Beta-Blocker, n (%)	30 (75.0%)	14 (77.8%)	16 (72.7%)	1.000	11 (78.6%)	19 (73.1%)	1.000
ACE-I or ARB, n (%)	28 (70.0%)	16 (88.9%)	12 (54.5%)	**0.035**	13 (92.9%)	15 (57.7%)	**0.030**
MRA, n (%)	20 (50.0%)	13 (72.2%)	7 (31.8%)	**0.025**	9 (64.3%)	11 (42.3%)	0.320
Diuretics, n (%)	39 (97.5%)	18 (100.0%)	21 (95.5%)	1.000	14 (100.0%)	25 (96.2%)	1.000
Furosemide dose, mg (IQR)	160 (78–250)	160 (80–250)	160 (40–250)	0.492	163 (80–330)	160 (75–250)	0.492
Electrolytes							
Serum sodium, mmol/l (IQR)	136 (134–139)	138 (135–140)	136 (132–137)	0.066	137 (135–139)	136 (134–139)	0.376
Serum potassium, mmol/l (IQR)	4.29 (3.93–4.60)	4.33 (4.08–4.59)	4.17 (3.85–4.70)	0.657	4.32 (4.08–4.50)	4.23 (3.85–4.73)	0.812
Serum calcium, mmol/l (IQR)	2.36 (2.22–2.43)	2.37 (2.28–2.44)	2.35 (2.20–2.41)	0.396	2.36 (2.22–2.43)	2.36 (2.22–2.42)	0.989
Serum phosphate, mmol/l (IQR)	1.31 (1.06–1.83)	1.27 (1.08–1.83)	1.38 (1.05–1.82)	0.861	1.27 (1.11–1.92)	1.34 (1.05–1.82)	0.685
Others							
C-reactive protein, mg/dl (IQR)	0.85 (0.51–1.84)	0.85 (0.51–1.53)	0.93 (0.51–3.14)	0.527	0.72 (0.34–1.16)	1.07 (0.52–3.14)	0.190
NT-proBNP, pg/ml (IQR)	17359 (8264–27145)	10226 (5621–29261)	19076 (11008–24965)	0.312	10446 (5621–29261)	18437 (8602–26753)	0.528
hsTnT, ng/ml (IQR)	0.096 (0.061–0.135)	0.063 (0.040–0.120)	0.111 (0.081–0.139)	**0.045**	0.083 (0.053–0.159)	0.096 (0.073–0.130)	0.624
Hemoglobin, g/dl (IQR)	11.0 (9.8–12.1)	11.5 (10.2–12.2)	10.3 (9.2–12.0)	0.119	11.2 (10.2–11.8)	10.7 (9.2–12.2)	0.392
Leukocyte count, G/l (IQR)	6.04 (5.46–7.22)	5.80 (5.15–6.95)	6.53 (5.56–7.74)	0.140	5.83 (5.15–7.04)	6.09 (5.56–7.54)	0.424
Albumin, g/L (IQR)	37.3 (34.4–41.3)	40.4 (37.1–42.2)	36.0 (33.7–39.9)	**0.045**	38.6 (35.8–41.4)	36.9 (34.2–41.0)	0.440
Uric acid, mg/dL (IQR)	8.30 (6.35–9.96)	9.50 (8.20–11.40)	7.05 (5.20–9.20)	**0.008**	9.75 (8.40–11.00)	7.35 (5.20–9.20)	**0.011**
AP, U/l (IQR)	114 (85–163)	111 (94–139)	130 (82–205)	0.465	114.0 (96.5–151.0)	112.5 (81.5–170.0)	0.679
AST, U/l (IQR)	20 (16–25)	18 (16–22)	25 (17–29)	0.106	20 (17–22)	20 (15–29)	0.624
ALT, U/l (IQR)	14 (10–18)	14 (10–15)	16 (10–25)	0.190	13 (10–15)	15 (11–25)	0.162
GGT, U/l (IQR)	109 (60–195)	123 (88–197)	95 (48–156)	0.299	134 (88–199)	98 (49–156)	0.279
BChE, kU/l (IQR)	3.55 (2.81–4.21)	3.94 (3.17–4.17)	3.03 (2.33–4.26)	0.180	3.94 (3.29–5.03)	3.03 (2.33–4.15)	0.071

IQR–interquartile range; BMI–body mass index; MLHFQ–Minnesota Living with Heart Failure Questionnaire; CMP—Cardiomyopathy; CAD carotic artery disease; TIA–transitory ischemic attack; PAD–peripheral artery disease; COPD–chronic obstructive pulmonary disease; PM–pacemaker; ICD–intracardiac defibrillator; CRT–cardiac resynchronization therapy; ACE–angiotensin converting enzyme; ARB–angiotensin receptor blocker, MRA–mineralocorticoid receptor antagonist; GFR–glomerular filtration rate; NT-proBNP–N-terminal B-type natriuretic peptide; hsTNT–high sensitive cardiac troponin T; AP–alkaline phosphatase; AST–aspartate transaminase; ALT–alanine transaminase; GGT–gamma-glutamyl transferase; BChE–butyryl-cholinesterase.

**Table 2:** Baseline echocardiographic parameters of the peritoneal dialysis patient cohort (n = 40) and comparison of variables for the subgroups with successful treatment and 2 years survival. Continuous variables are given as medians and inter-quartile ranges (IQR), counts are given as numbers and percentages. Variables were compared by the means of the Mann-Whitney-U test or the Chi-square test.

	Baseline (n = 40)	PD Success (n = 18)	No PD Success (n = 22)	p-value	2a Survival (n = 14)	No 2a survival (n = 26)	p-value
LVEF, % (IQR)	29 (23–36)	27 (23–35)	30 (23–39)	0.476	26 (22–31)	30 (27–39)	0.071
LVF sq <35%, n (%)	33 (82.5%)	15 (83.3%)	18 (81.8%)	1.000	12 (85.7%)	21 (80.8%)	1.000
RVF sq moderately or severely reduced, n (%)	33 (82.5%)	17 (94.4%)	16 (72.7%)	**0.016**	12 (85.7%)	21 (80.7%)	0.065
RV FAC,—(IQR)	27.1 (22.2–34.8)	25.0 (22.2–30.6)	30.3 (25.7–39.6)	0.231	22.6 (19.5–26.8)	32.7 (26.3–39.8)	**0.001**
TAPSE, mm (IQR)	11 (9–13)	10 (8–12)	12 (10–15)	0.157	11 (9–15)	11 (9–12)	0.897
LV Diastolic dysfunction pseudonomral or restrictive, n (%)	27 (93.0%)	11 (91.7%)	16 (94.2%)	0.957	9 (100.0%)	18 (90.0%)	0.201
Mitral valve regurgitation mild, n (%)	12 (30.0%)	5 (27.8%)	7 (31.8%)	0.966	4 (28.6%)	8 (30.8%)	0.894
Mitral valve regurgitation moderate, n (%)	18 (45.0%)	9 (50.0%)	9 (40.9%)		7 (50.0%)	11 (42.3%)	
Mitral valve regurgitation severe, n (%)	10 (25.0%)	4 (22.2%)	6 (27.3%)		3 (21.4%)	7 (26.9%)	
Tricuspid valve regurgitation mild, n (%)	3 (7.5%)	0 (0.0%)	3 (13.6%)	**0.035**	0 (0.0%)	3 (11.5%)	0.402
Tricuspid valve regurgitation moderate, n (%)	10 (25.0%)	3 (16.7%)	7 (31.8%)		4 (28.6%)	6 (23.1%)	
Tricuspid valve regurgitation severe, n (%)	27 (67.5%)	15 (83.3%)	12 (54.5%)		10 (71.4%)	17 (65.4%)	
Estimated sPAP, mmHg (IQR)	59 (49–71)	68 (56–72)	56 (48–65)	**0.039**	65 (50–71)	56 (48–65)	0.244

IQR–interquartile range; LVEF–left ventricular ejection fraction; LVF sq–semiquanitative assessment of left ventricular function; RVF sq–semiquantitative assessment of right ventricular function; RV FAC–right ventricular fractional area change; TAPSE—tricuspid annular plane systolic excursion; LV–left ventricular; sPAP–systolic pulmonary arterial pressure.

**Table 3:** Baseline kidney related and peritoneal dialysis related parameters of the peritoneal dialysis patient cohort (n = 40) and comparison of variables for the subgroups with successful treatment and 2 years survival. Continuous variables are given as medians and inter-quartile ranges (IQR), counts are given as numbers and percentages. Variables were compared by the means of the Mann-Whitney-U test or the Chi-square test.

	Baseline (n = 40)	PD Success (n = 18)	No PD Success (n = 22)	p-value	2-years Survival (n = 14)	No 2-years survival (n = 26)	p-value
Kidney related parameters							
Urinary output / 24h, ml (IQR)	1350 (750–1975)	1750 (950–2300)	1040 (450–1500)	**0.024**	1750 (950–2300)	1200 (450–1560)	**0.027**
Proteinuria, g/24 h (IQR)	0.09 (0.00–0.26)	0.08 (0.00–0.17)	0.12 (0.00–0.42)	0.427	0.11 (0.00–0.24)	0.08 (0.00–0.38)	0.834
Serum creatinine, mg/dl (IQR)	2.89 (1.93–4.34)	2.99 (1.71–4.08)	2.87 (2.00–4.50)	0.459	2.99 (1.98–3.76)	2.87 (1.88–4.47)	0.644
eGFR, mL/min/1.73 m^2^ (IQR)	19.44 (10.86–33.93)	20.26 (15.54–38.77)	17.37 (10.52–26.43)	0.132	20.26 (15.54–36.59)	17.37 (10.42–32.69)	0.123
24hGFR, mL/min/1.73 m^2^ (IQR)	9.67 (6.24–19.30)	14.74 (7.71–25.33)	8.83 (3.41–16.33)	**0.027**	14.74 (8.48–25.33)	9.37 (3.41–17.00)	**0.045**
Blood urea nitrogen, mg/dl (IQR)	62.55 (36.60–104.50)	61.50 (37.20–110.10)	64.05 (32.20–92.10)	0.737	77.45 (45.80–121.10)	58.55 (31.30–95.10)	0.243
pH (IQR)	7.37 (7.32–7.40)	7.38 (7.35–7.40)	7.37 (7.29–7.40)	0.346	7.36 (7.34–7.39)	7.38 (7.31–7.41)	0.846
HCO3-, mmol/L (IQR)	23.85 (21.50–26.40)	23.85 (21.90–26.60)	23.45 (20.10–26.35)	0.573	23.75 (21.90–26.60)	23.95 (20.75–26.35)	0.687
PD related parameters							
Ascitic fluid volume, ml (IQR)	1110 (0–2000)	2000 (1100–3000)	400 (0–2000)	**0.017**	1750 (60–2500)	840 (0–2000)	0.266
PET, D/P creatinine 4 h (IQR)	0.74 (0.73–0.86)	0.83 (0.73–0.88)	0.74 (0.68–0.84)	0.160	0.83 (0.69–0.88)	0.74 (0.74–0.86)	0.434
Help with PD “no assistance”, n (%)	23 (57.5%)	17 (94.4%)	6 (27.3%)	**<0.001**	14 (100.0%)	9 (34.6%)	**<0.001**
Help with PD type “partial assistance”, n (%)	3 (7.5%)	0 (0.0%)	3 (13.6%)		0 (0.0%)	3 (11.5%)	
Help with PD “full assistance”, n (%)	14 (35.0%)	1 (5.6%)	13 (59.1%)		0 (0.0%)	14 (53.8%)	

IQR–interquartile range; GFR–glomerular filtration rate;, HCO3—serum bicarbonate; D/P—dialysate—to plasma ratio; PET–peritoneal equilibration test.

### PD characteristics

PD catheter was inserted using the Seldinger technique in 25 patients, and surgically (laparotomy) in 15 patients (local anesthesia with or without sedoanalgesia, n = 10, general anesthesia, n = 5). In patients with ascites a stepwise drainage of intraperitoneal fluid (1–3 liters/day, depending on blood pressure) was started immediately after catheter implantation. In patients with significantly impaired renal function one single nocturnal exchange with icodextrin-containing PD fluid or automated peritoneal dialysis (APD) with the use of a cycler was started (depending on clinical requirements). Fourteen patients started with drainage of ascites, 5 patients with a single nocturnal exchange with icodextrin and 21 patients started with APD. Five patients used amino acid-containing PD fluid as part of the PD prescription, 3 patients were treated with oral nutritional supplements. During the observation period treatment was intensified in 8 patients whereas dialysis dose could be decreased in 5 patients and remained unchanged in 27 patients. Twenty-six (65%) patients needed assistance for PD.

### PD associated complications

In one of the 4 patients with catheter tip migration change of the PD catheter was required. One patient died due to cardiac arrest immediately after catheter implantation unrelated to the procedure. Two patients developed hydrothorax due to pleuroperitoneal communication without requirement of intervention. One of these patients was successfully bridged to LVAD implantation. The other patient had to be transferred to intermittent hemodialysis. Repeated hypotensive episodes occurred during this treatment. Peritonitis rate was 1 episode/37.3 patient months.

### Clinical course and overall survival

In 4 patients implantation of an LVAD had to be primarily displaced because of the poor clinical condition and impairment of RV function. After start of PD the clinical condition improved significantly in all of these patients and the intervention could be performed after 15 days, 33 days, 11 months and 13 months, respectively. Three of these patients underwent heart transplantation thereafter. Another two patients without LVAD underwent heart transplantation 4 months and 19 months after start of PD. All other patients were not suitable candidates for heart transplantation (based on age or co-morbidities) or LVAD (based on severe RV function and impaired kidney function). Patient overall survival was 55.0% (22 patients) at 1 year, 35.0% (14 patients) at 2 years and 27.5% (11 patients) at 3 years. Reasons for death are summarized in *Table 4*.

**Table 4:** Reasons for death at 3 years (n = 29).

Reasons for death (n = 29)	
Worsening of cardiac failure, n (%)	14 (48%)
Sudden death, n (%)	4 (14%)
Myocardial infarction, n (%)	1 (3%)
Sepsis, n (%)	6 (21%)
Pneumonia, n (%)	2 (7%)
Bowel necrosis, n (%)	1 (3%)
Intracranial bleeding, n (%)	1 (3%)

### Hospitalization

**Fig 1:**
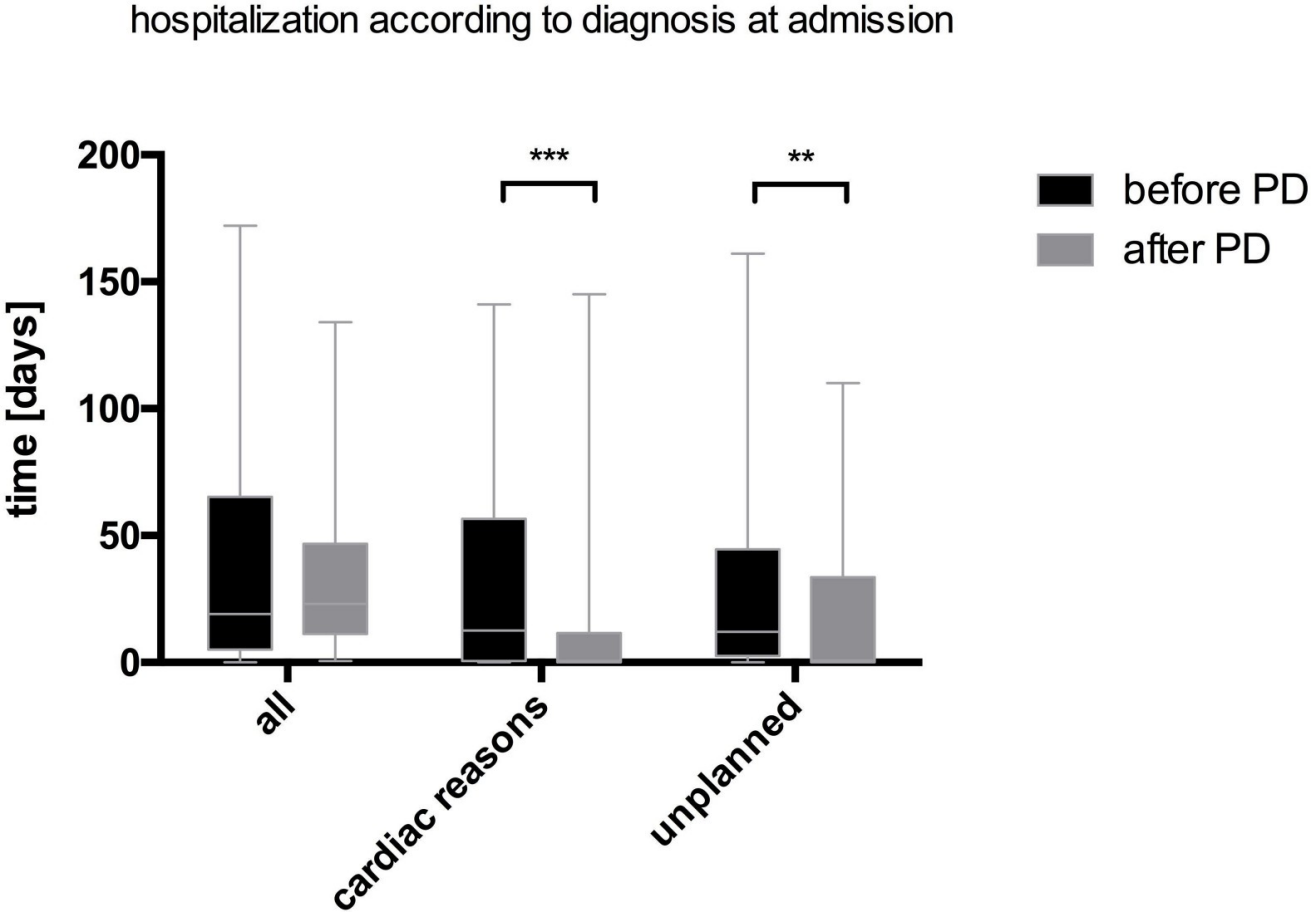
Hospitalization days before and after the initiation of PD (n = 40). Variables are displayed as mean and SEM. Differences between the variables were compared using the Wilcoxon test. ** indicates statistical significance with p<0.01 and *** with p<0.001.

After start of PD the number of hospitalization days due to cardiac reasons, i.e. the primary endpoint, declined significantly compared to the period before starting the therapy [13 (IQR 1–53) days vs. 1 (IQR 0–12) days, p<0.001]. Similarly, the number of hospitalization days due to unplanned reasons declined significantly with PD initiation [12 (IQR 3–44) days vs. 1 (IQR 0–33) days, p = 0.007] (*Table 5, Fig 1*). The overall number of hospitalization days before and after start of PD did not differ significantly [19 (IQR 5–62) days vs. 23 (IQR 12–47) days, p = 0.878]. However, it must be noted that after the initiation of PD two patients were admitted to the hospital for long inpatient treatment owed to non-PD related or cardiac reasons (59 days and 65 days stay due to a vertebral fracture and calciphylaxis, respectively).

**Table 5:** Hospitalization days for patients on peritoneal dialysis (PD) before and after starting the therapy (n = 40). Total hospitalization days, hospitalization days due to cardiovascular (CV) reasons and unplanned hospitalization days are given as medians and inter-quartile ranges (IQR). Differences between hospitalization days before and after the initiation of PD are calculated using the Wilcoxon test.

	Before the initiation of PD	After the initiation of PD	P-value
Total Hospitalization days, n (IQR)	19 (5–62)	23 (12–47)	0.878
Hospitalization due to Cardiac reasons, n (IQR)	13 (1–53)	1 (0–12)	**<0.001**
Unplanned hospitalization days, n (IQR)	12 (3–44)	1 (0–33)	**0.007**

IQR–interquartile range. Fonts in bold indicate statistical significance.

### Subgroup analysis for patients with treatment success

Eighteen (45.0%) patients fulfilled the predefined criteria for treatment success. There were no significant differences at baseline between successfully and unsuccessfully treated patients in age, gender, body mass index, quality of life, comorbidities, furosemide dose or NT-proBNP levels. Patients with treatment success had better kidney function mirrored by a higher 24-h-GFR [14.74 (IQR 7.71–25.33) ml/min/1.73m^2^ vs 8.83 (IQR 3.41–16.33) ml/min/1.73m^2^, p = 0.027] and higher urinary volume [1750 (IQR 950–2300) ml vs 1040 (IQR 450–1500) ml, p = 0.024], and were less frequently depending on assistance for PD (p<0.001) (*Table 3*). With regards to backward failure, successfully treated patients had a higher amount of ascites at baseline [2000ml (IQR 1100–3000) ml vs 400ml (IQR 0–2000) ml, p = 0.017], more severe tricuspid regurgitation (p = 0.035), more severely impaired right ventricular function (semiquantitative assessment, p = 0.016) and higher values of systolic pulmonary artery pressure [68 (IQR 56–72) mmHg vs 56 (IQR 48–65) mmHg, p = 0.039]. Furthermore, they had lower troponin T concentrations, higher serum albumin and uric acid levels and more frequent temporary/persistent atrial fibrillation. Finally, among patients with successful treatment, the administration of RAS antagonists and mineralocorticoid receptor antagonists could be maintained in a higher percentage (*Tables 1–3*).

### Subgroup analysis for 2-year survivors

A total of 14 (35.0%) patients were alive after 2 years of treatment initiation. These patients had higher baseline 24h-GFR, higher baseline urinary volume, and less frequent assistance for PD compared with those who did not survive. Furthermore, 2-year survivors had a lower heart rate, a higher serum uric acid concentration and were more frequently treated with RAS antagonists than non-survivors. In 2-year survivors RVF tended to be impaired more frequently in the semiquantitative assessment, whereas RV FAC was significantly reduced (*Tables 1–3*). The results of the Cox regression analysis are shown in S1 Table. Higher urinary output and increased serum sodium levels were associated with better 2-years survival in the univariate model as well as after adjustment to NT-proBNP and age.

### Longitudinal changes in patients with treatment success and survival ≥2 years

**Fig 2:**
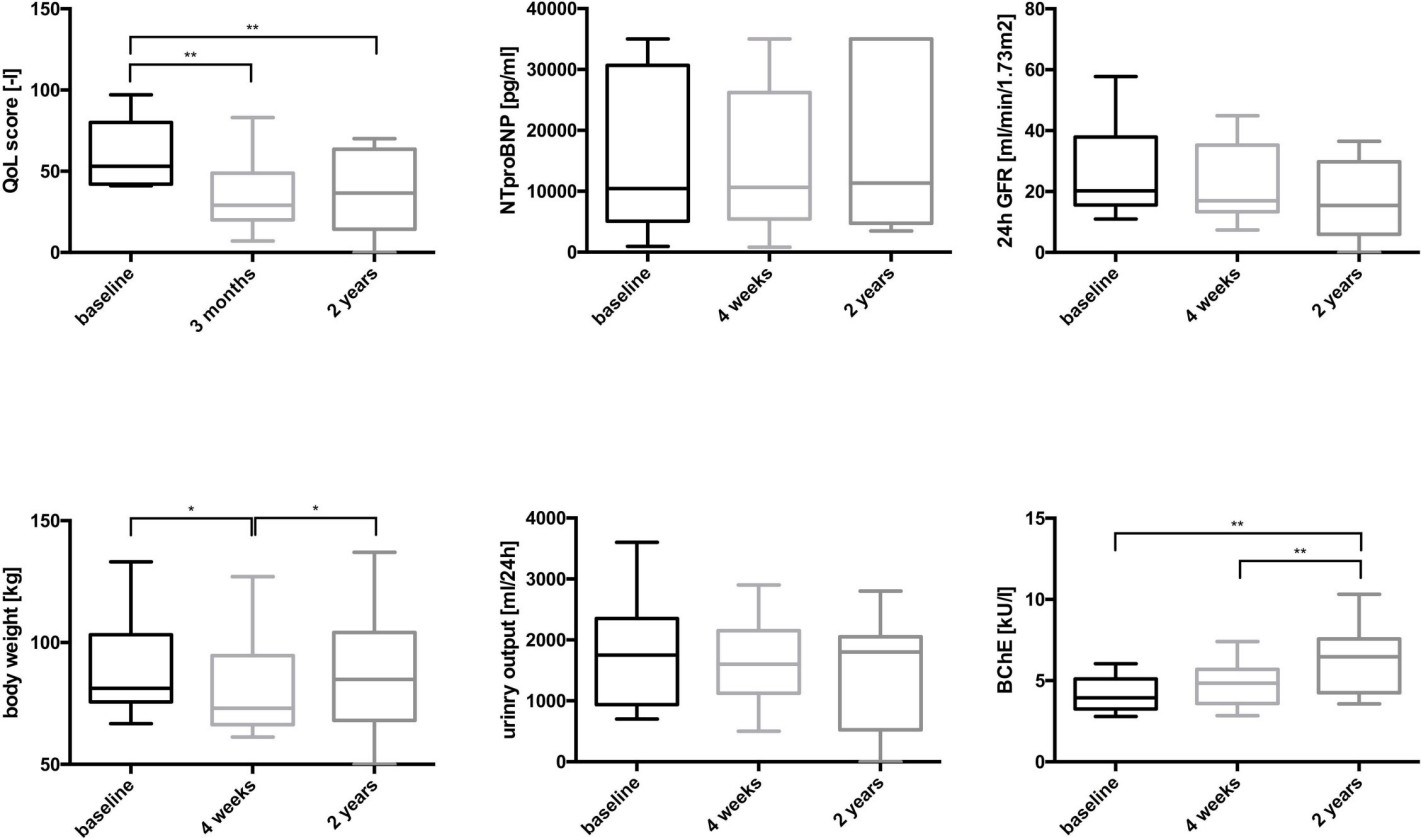
Changes of parameters after initiation of PD in patients with an overall survival ≥ 2 years (n = 14). Variables are displayed as Tukey boxplots. Differences between the variables were compared using the Wilcoxon test. * indicates statistical significance with p<0.05 and ** with p<0.01.

Parameters of clinical interest at baseline, 4 weeks and at follow-up of PD treatment for patients with treatment success and ≥ 2 years survival are shown in *Table 6*. The course of body weight, urinary output, 24h-GFR, NT-proBNP, Butyryl-cholinesterase (BchE) and the quality of life score for successfully treated patients are additionally displayed in *Fig 2*.

**Table 6:** Biometric variables for patients with peritoneal dialysis with successful therapy (n = 18) or surviving ≥ 2 years (n = 14). Variables are given as medians and inter-quartile ranges (IQR). Differences between the variables days before and after the initiation of PD are calculated using the Wilcoxon test.

	Successful treatment baseline	Succesful treatments 4 weeks	Successful treatment 1 year	P-value	2-year survival baseline	2-year survival 4 weeks	2-year survival 2 years	P-value
Body weight, kg (IQR)	83.1 (68.6–95.5)	73.0 (66.0–94.0)	83.5 (66.0–97.4)	**0.011**	81.2 (78.0–97.8)	73.0 (66.5–91.1)	84.9 (68.0–104.1)	**0.020**
nPCR, g/kg/d (IQR)	0.92 (0.61–1.08)	1.11 (0.84–1.39)	0.87 (0.73–1.13)	0.145	0.92 (0.71-1-11)	1.29 (0.91–1.41)	0.84 (0.74–0.95)	0.122
Urinary output, ml (IQR)	1900 (1400–2200)	1600 (1050–1920)	1900 (1400–2100)	0.689	1750 (950–2300)	1600 (1200–2100)	1800 (600–1900)	0.773
eGFR, ml/min/1.7m^2^ (IQR)	18.61 (15.54–36.59)	21.36 (13.69–36.46)	17.32 (7.84–30.88)	0.458	20.26 (15.54–36.59)	16.98 (13.49–34.01)	15.45 (6.03–28.17)	0.273
NT-proBNP, pg/ml (IQR)	10011 (5621–29261)	7420 (5147–16482)	6583 (3154–16888)	0.920	10446 (5621–29261)	10662 (5728–17461)	11344 (4765–35001)	0.918
BChE, kU/l (IQR)	3.94 (3.17–4.15)	4.33 (3.35–5.40)	5.10 (4.61–7.52)	**0.001**	3.94 (3.29–5.03)	4.84 (3.84–5.49)	6.47 (4.25–7.57)	**0.003**
Albumin, g/l (IQR)	40.4 (37.1–42.2)	36.3 (33.6–41.1)	37.2 (34.4–43.2)	0.223	38.6 (35.8–41.4)	36.3 (33.6–41.1)	37.6 (31.1–38.0)	0.273
Total cholesterol, mg/dl (IQR)	126 (108–147)	188 (145–208)	181 (150–232)	**<0.001**	126 (106–151)	162 (143–208)	170 (147–214)	**0.002**
Transferrin, mg/dl (IQR)	280.3 (251.7–322.7)	271.6 (231.9–302.6)	266.1 (244.6–301.9)	0.145	272.5 (251.7–300.8)	271.6 (238.2–288.9)	256.3 (223.9–267.6)	0.121
QoL,—(IQR)	57 (43–83)	29 (21–52)^#^	25 (16–50)	**<0.001**	53 (42–77)	29 (20–42)^#^	37 (16–62)	**0.016**

IQR–interquartile range; nPCR–normalized protein catabolic rate; GFR–glomerular filtration rate; NT-proBNP–N-terminal B-type natriuretic peptide; BChE–butyryl-cholinesterase; QoL–quality of life. Fonts in bold indicate statistical significance.

# 3 months

In patients with treatment success quality of life improved significantly after 3 months [57 (IQR 43–83) vs 29 (IQR 21–52); p = 0.002] and remained stable at 1 year [57 (IQR 43–83) vs 25 (IQR 16–50); p<0.001]. Two-year survivors similarly improved with QoL score at 3 months [53 (IQR 42–77) vs 29 (IQR 20–42); p = 0.015] and remained improved at 2 years [53 (IQR 42–77) vs 33 (IQR 16–62); p = 0.016]. There was only a temporary decrease of body weight at 4 weeks. BChE increased significantly during the whole observation period. In contrast, there were no significant changes in NT-proBNP, 24h-GFR or daily urinary volume at 4 weeks, 1 year or 2 years after start of PD for these patients. Because of the solely temporary decrease of body weight several nutritional parameters were additionally analyzed in patients with treatment success and patients who survived at least 2 years. Serum albumin, serum transferrin and nPCR did not change significantly, but cholesterol levels increased significantly after start of PD (Table 6).

## Discussion

In our population of patients with right heart failure we could show a significant decline of hospitalization days both due to cardiac and unplanned reasons. For patients with successful treatment quality of life improved significantly after 3 months of treatment and remained enhanced during the rest of the observation period. Using a combined endpoint which included not only the mere survival time but a reduction in hospitalization days and also an improvement in quality of life we found that patients with extended ascites, higher systolic pulmonary artery pressure, more marked impairment of right ventricular function and tricuspid valve insufficiency as well as those who could perform PD without assistance most benefited from this therapy. Similar factors were associated with 2 year survival.

Our study differs from previous studies in several points. Many published papers were single case reports. Only 4 studies included ≥ 40 patients [20–23], whereas two of these 4 studies were retrospective in nature [21, 22], and the prospective cohort studies included mainly patients treated with acute temporary high-volume PD [23] or intermittent in-center PD [20]. In contrast to our study, robust inclusion criteria in previous reports remained unclear and the definition of end-stage heart failure was left to the discretion of the investigators. The range of survival between 50% and 100% at 1 year reported in PD patients with refractory heart failure in previous papers reflects a wide variation in morbidity and types of heart disease of the studied patient population (12, 16). In contrast to that, we have focused on the predominance of backward failure and included only patients with objective signs of RHF, mirroring this pathophysiology.

Patients suffering from right heart failure and kidney dysfunction are regarded to have the worst prognosis [24] and are not eligible for LVAD implantation. The survival rates in this study (35% after 2 years) are in line with the data reported in patients without PD [24]. However, there are several aspects indicating that we have included a patient population with a comparably more advanced disease. The most important prognostic marker, NT-proBNP, differed tremendous between the study of Dini et al. [24] and our data (2644pg/ml vs. 17359pg/ml, respectively). This is clinically important even when considering that kidney function was markedly impaired in our study population. NT-proBNP levels found in the present study also exceed three times the levels of LVAD patients [25]. Furthermore, the percentage of ICD/CRT patients (52.5%/35.0%) was markedly higher than in some other studies. As depicted in the demographic data, the background therapy was already up-titrated to the recommended dosages. This is an important factor, as we have become aware, that invasive and cost expensive methods should only be initiated in the case of optimal therapy [26].

In agreement with previous studies, our data show a significant decrease in hospitalization and an improvement of quality of life after the initiation of PD, confirming that these findings are equally true for patients with refractory right heart failure. Furthermore, we have found that not only hospitalizations due to cardiac reasons but also unplanned admissions declined significantly after PD initiation. Several other studies describe an improvement of quality of life in PD patients with refractory heart failure using NYHA functional classes. With the Minnesota Living with Heart Failure Questionnaire our study applied a multidimensional disease-specific tool for assessing quality of life in heart failure patients [15, 27].

While our study was not designed to prove that PD provides survival benefit in the respective patient group, hospitalizations as well as improvement of quality of life over time are excellent surrogates. Therefore, we defined a combined endpoint incorporating these surrogates as an indicator of favorable therapy response. Characteristics of patients, who are more likely to benefit from PD, included a higher GFR at baseline, underscoring previous data that such an intervention should not be started too late [28]. Moreover, our findings support the hypothesis that patients with more pronounced backward failure, i.e. patients with higher systolic pulmonary artery pressure, more marked impairment of right ventricular function, tricuspid valve insufficiency and extensive ascites would profit more from PD. Interestingly, patients who cannot perform PD without assistance, show less benefit from this treatment. While these patients reflect an especially sick subpopulation this finding may also underscore the fact that self-reliant behavior is an important factor, even in end-stage heart failure. Interestingly heart failure severity markers as NT-proBNP and LVEF were not associated with adverse outcome. However, higher urinary output and elevated serum sodium levels indicated better prognosis in the multivariate Cox regression analysis. It can be hypothesized that therapy refractoriness with correspondingly high and probably varying NT-proBNP levels as well as impaired kidney function limits the predictive ability of NT-proBNP as a biomarker in this patient population.

The initiation of PD in patients with right heart failure was associated with a marked decline of body weight at 4 weeks after start of therapy, indicating decongestion. As another marker of decongestion and improvement of backward failure BChE, a sensitive marker of functional liver congestion, increased significantly. However, kidney function did not change. This is remarkable, as an improvement of kidney function was suggested during decongestion [5]. In contrast to previous studies, median baseline GFR in our study was 9–10 ml/min/1.73 m^2^ and therefore highly impaired. It is likely that our patients displayed a more advanced stage of kidney disease with already morphologic rather than only functional impairment. Accordingly, some previous papers also reported no improvement of residual renal function in PD patients with refractory heart failure [9]. Besides, our data confirm previous studies reporting that eGFR does not accurately reflect renal function in several other patient populations with kidney diseases [29–33]. Most importantly, we confirm that in patients with medical conditions which are associated with reduced muscle mass (as usual in refractory heart failure) eGFR markedly overestimates true GFR [34, 35] and should, therefore, be used with caution in these patients. Body weight of patients with treatment success showed only a temporary decline after start of PD, reaching values at 1 year which were comparable with those at baseline. Based on albumin, transferrin and nPCR we could confirm neither an improvement nor impairment of protein intake/protein metabolism. However, cholesterol levels increased significantly after start of PD. Interestingly, Fröhlich et al reported an increase of fat mass (not lean body mass) in patients with refractory heart failure treated with PD [36].

As limitation of this study it should be considered that this is a non-randomized trial without a control group. However, it remains difficult to randomize patients with refractory heart failure before defining the right target population, which was the main intention of our project. The patient number is comparable to other studies, but still quite low. Therefore, sophisticated statistics is not appropriate, but certainly our data can stimulate the investigation of larger cohorts.

In conclusion, PD is associated with a decrease in hospitalization and improvement of quality of life in patients with refractory right heart failure. Our data suggest that the patients most suitable for PD have high values of systolic pulmonary artery pressure, a marked impairment of RV function, a marked tricuspid regurgitation, a large amount of ascites and some degree of residual renal function and are autonomous concerning the planned therapy.

## Supporting information

S1 Table Cox regression analysis on clinical characteristics in therapy refractory HF patients treated by PD—Unadjusted and adjusted effects on 2 years mortality (n = 40, events = 22). (DOCX)
