# Supplementary material for: Parameters associated with therapeutic response using peritoneal dialysis for therapy refractory heart failure and congestive right ventricular dysfunction
Source: PLoS One. 2018 Nov 19;13(11):e0206830. doi: 10.1371/journal.pone.0206830 (PMC6242305; doi:10.1371/journal.pone.0206830)
Supplement: S1 Table — (DOCX) [file pone.0206830.s001.docx]

**Supporting Information**

**S1 Table. Cox regression analysis on clinical characteristics in therapy refractory HF patients treated by PD - unadjusted and adjusted effects on 2 years mortality (n=40, events=22).** Cox proportional hazard models for different variables are shown. Hazard ratios (HR) refer to a 1-IQR increase in continuous variables. In the multivariate model HRs are adjusted (adj.) for age and NT-proBNP.

| Variables | IQR | Crude HR (95%CI) | P-value | Adj. HR^1^ (95%CI) | P-value |
| --- | --- | --- | --- | --- | --- |
| NT-proBNP, pg/ml | 18881 | 1.20 (0.62-2.31) | 0.584 | - | **-** |
| LVEF, % | 13 | 1.57 (0.90-2.78) | 0.115 | - | - |
| Urinary output / 24h, ml | 1225 | 0.31 (0.15-0.64) | **0.002** | 0.29 (0.13-0.65) | **0.003** |
| 24hGFR, ml/min/1.73m^2^ | 13.06 | 0.42 (0.18-1.01) | 0.052 | - | **-** |
| eGFR, ml/min/1.73m^2^ | 23.07 | 0.58 (0.25-1.35) | 0.207 | - | - |
| Serum Sodium mmol/l | 5 | 0.51 (0.28-0.93) | **0.028** | 0.50 (0.27-0.90) | **0.020** |
| BUN, mg/dl | 67.9 | 1.64 (0.31-1.31) | 0.637 | - | - |
| BUN/creatinine ratio | 14.67 | 0.55 (0.28-1.07) | 0.079 | - | - |
| BChE, kU/l | 1.40 | 0.61 (0.35-1.05) | 0.073 | - | - |
| Ascitic fluid volume, ml | 500* | 0.94 (0.82-1.08) | 0.938 | - | - |

^1^ HR adjusted to age, NT-proBNP; ^*^ arbitrary unity.

IQR – inter quartile range; NT-proBNP – N-terminal B-type natriuretic peptide; LVEF – left ventricular ejection fraction; GFR – glomerular filtration rate; eGFR – estimated GFR; BUN - blood urea nitrogen; BChE – butyryl-cholinesterase.
